# Supplementary material for: Persistent BK Polyomavirus Viruria Is Associated with Accumulation of VP1 Mutations and Neutralization Escape
Source: Viruses. 2020 Jul 29;12(8):824. doi: 10.3390/v12080824 (PMC7472262; doi:10.3390/v12080824)
Supplement: Supplementary file 1 [file viruses-12-00824-s001.zip › SupplementaryData/Supplementary_Figures.pdf]

Supplementary Figure 1

Calculation of ratio of infectious particles to pEGFP copies for pseudotype virus with different VP1 variants in HEK 293TT cells.

A) GFP and Hoechst fluorescence – one field per well.

B) %GFP^{+} cells counted from images in (A) as a function of quantity of PSV in pEGFP copies/cell

C) Intercept at 1% infected cells used to calculate ratio of infectious particles to number of genome copies in PSV prep

D) Inter-assay reproducibility of infectivity of gIb2$E^{72}E^{83}$ PSV. Expt 22/12/17 compared two independent quadruplicates set up in different plates. Expt 22/06/19 compared two independent PSV preps.

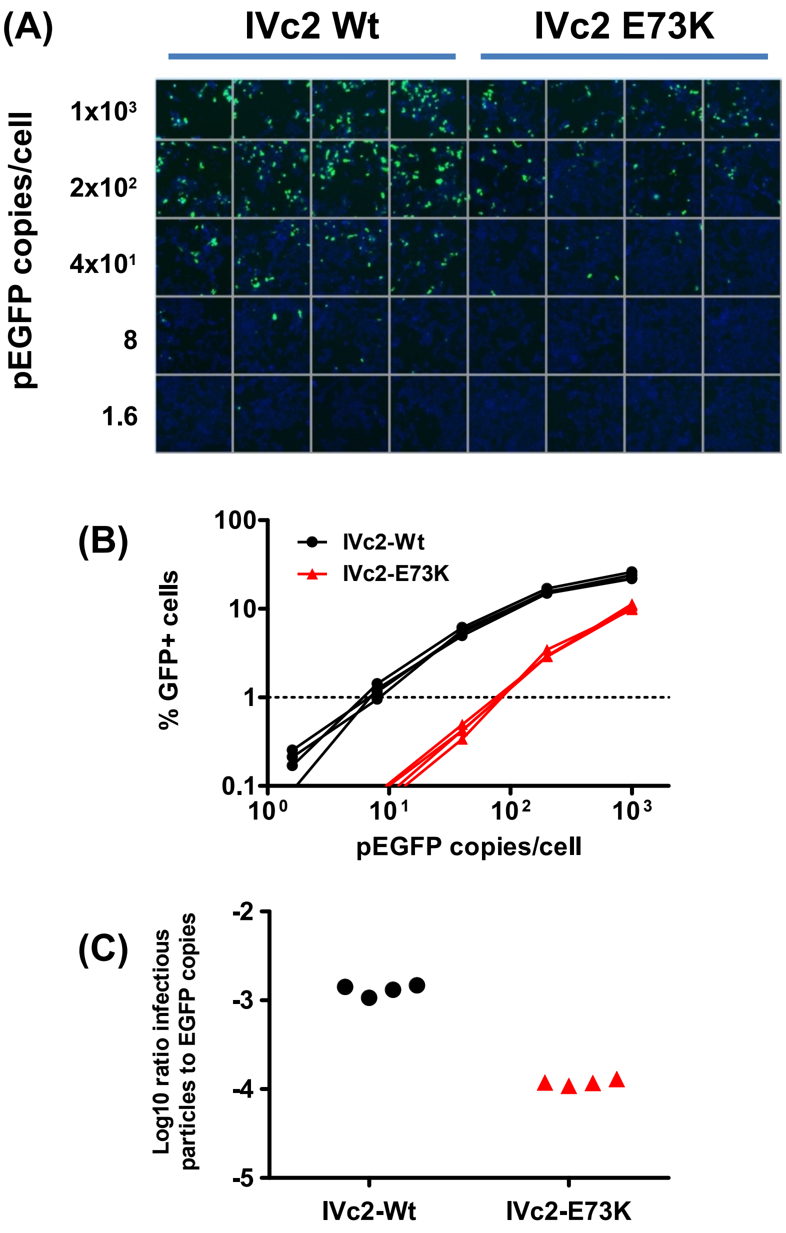

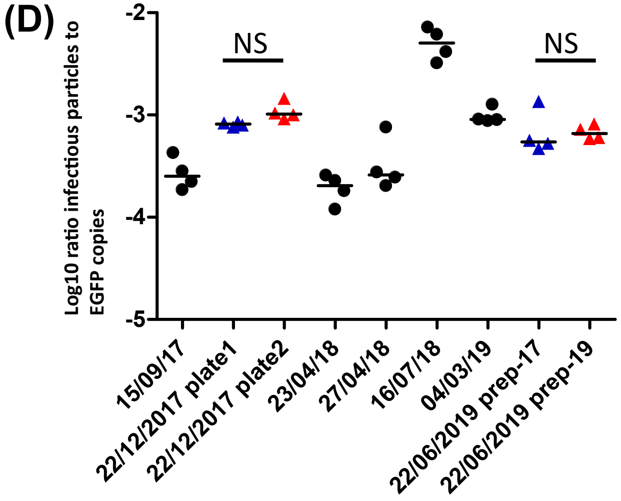

Supplementary Figure 2

TAg expression in Vero cells transfected with recircularized pBKV 35-1 plasmid carrying either Wild-type, A72V E73Q E82Q, or A72V E73K mutant VP1. 1x10[4] cells per well were transfected with 200 ng/well religated pBKV 35-1 using Lipofectamine 2000 reagent, then analyzed for Tag expression by immunofluorescence with mouse monoclonal anti-SV40 TAg (PAb416 clone, Abcam) followed by Alexa-488 conjugated goat anti-mouse IgG. Nuclei were counterstained with Hoechst 33342 and images were acquired on a Cellomics ArrayScan VTI HCS Reader. Cells transfected with uncut pBKV 35-1 can express Tag, but cannot release BKPyV particles, since the BamHI cloning site interrupts *VP1*. TAg expression over and above that observed in cells transfected with uncut pBKV 35-1 therefore represents secondary infections.
The *VP1* typing region was amplified from supernatants of two duplicate wells at d12 post-transfection and analyzed by Sanger sequencing. No reversion to wild-type *VP1* was observed.

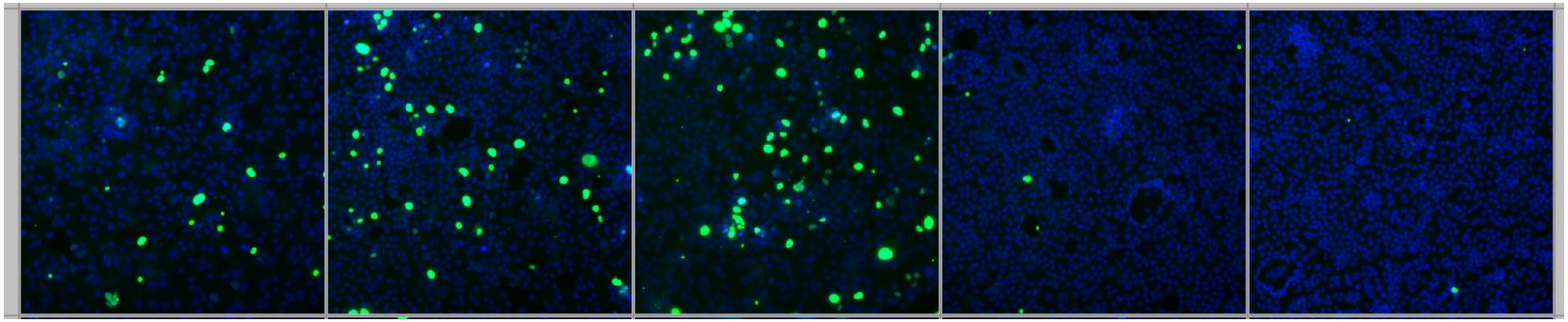

Wild-type | **A72V E73Q E82Q** | **A72V E73K** | **Wild-type pBKV 35-1, not cut, not religated** | **Negative control** **(Non-transfected)**

Species/Abbrv	*	*	*	*	*			*	*	*	*	*	*	*	*		*	*	*	*	*	*
1. VQQ-2_VPS1 --	S	L	K	L	S	V	Q	N	D	F	S	S	D	S	P	Q	R	K	M	L	P	C
2. VQQ-1_VPS1 --	S	L	K	L	S	V	Q	N	D	F	S	S	D	S	P	Q	R	K	M	L	P	C
3. VKE-2_VPS1 --	S	L	K	L	S	V	K	N	D	F	S	S	D	S	P	E	R	K	M	L	P	C
4. VKE-1_VPS1 --	S	L	K	L	S	V	K	N	D	F	S	S	D	S	P	E	R	K	M	L	P	C
5. BKMM-2_VPS1	S	L	K	L	S	A	E	N	D	F	S	S	D	S	P	E	R	K	M	L	P	C
6. BKMM-1_VPS1	S	L	K	L	S	A	E	N	D	F	S	S	D	S	P	E	R	K	M	L	P	C
7. BK MM - VP1	S	L	K	L	S	A	E	N	D	F	S	S	D	S	P	E	R	K	M	L	P	C

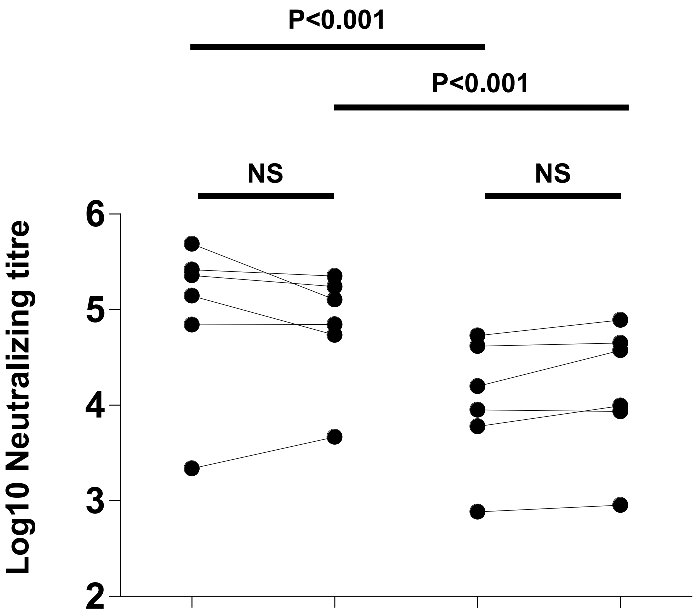

PSV	Ib2-EE	Ia	Ib2-EE	Ia
pEGFP copies/cell	$1x10^2$	$1x10^2$	$5x10^2$	$5x10^2$

Supplementary Figure 3

Increased pseudotype virus dose significantly reduces measured neutralizing titre

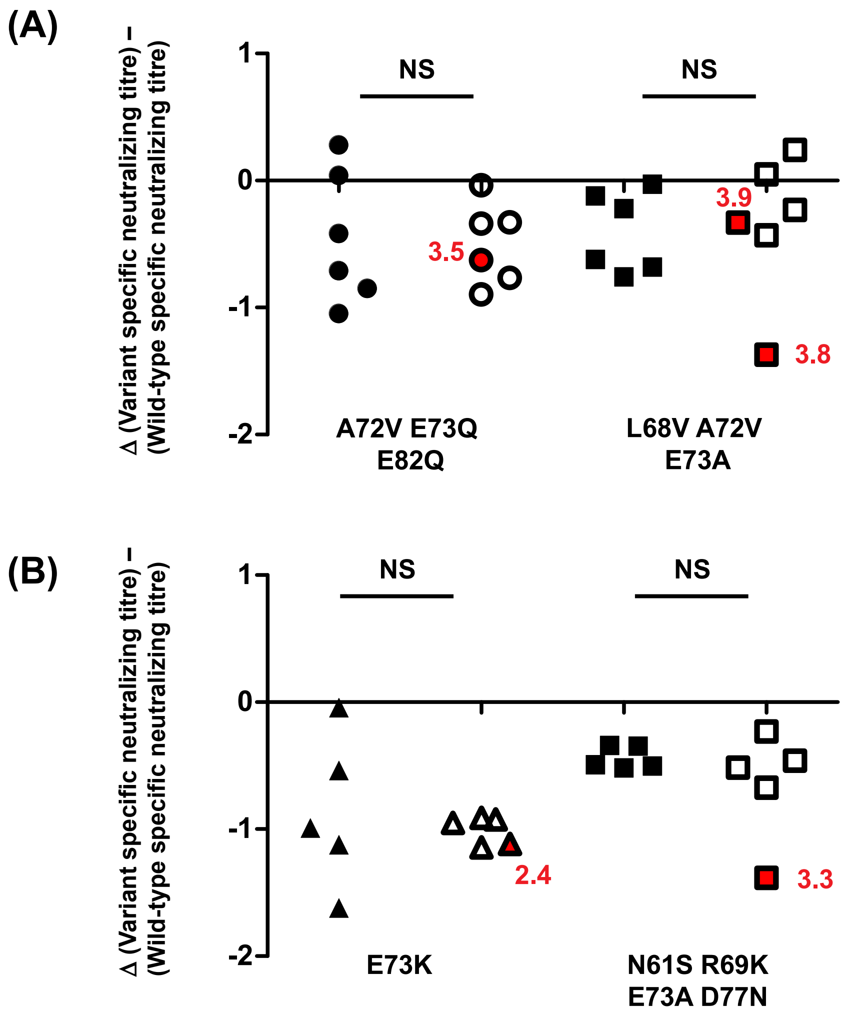

Supplementary Figure 4

Neutralization of PSV carrying VP1 variants in RS cells

Neutralizing titres in controller (n=6, filled symbols) and non-controller (n=6, open symbols) sera at 12 months post-KTx were measured against PSV carrying wild type VP1 and different VP1 variants. Experiments were performed in RS cells. For each serum, the log10 neutralizing titre against the wild-type virus was subtracted from the log10 neutralizing titre against the VP1 variant measured at the same PSV dose, and the result is plotted for each PSV variant. (A) genotype Ib2 variants; (B) genotype IVc2 variants. Red symbols indicate the cognate non-controller serum for each VP1 variant. Input PSV doses were 500 pEGFP copies/cell.

Infectivity of Ib2-N^{69}Q^{82} and Ib2-N^{60}N^{69}V^{72}Q^{82} PSV in RS cells was too low for reliable determination of neutralizing titres against these variants in RS cells.

Supplementary Figure 5

Cumulative effect of VP1 mutations on neutralization escape

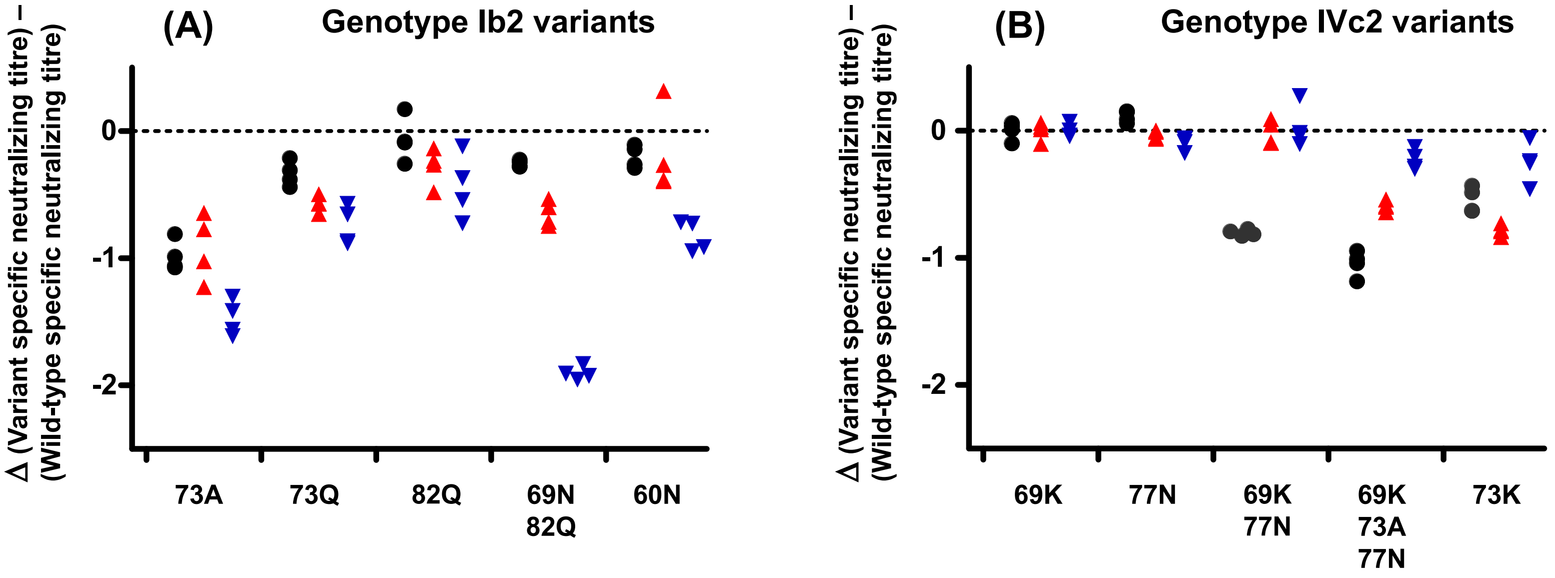

Patient	60	68	69	72	73	75	82
● 3.5	D	L	M	V	Q	N	Q
▲ 3.8	D	V	K	V	A	D	E
▼ 3.4	N	L	N	V	E	D	Q

Patient	61	62	64	69	72	73	75	77
● 3.3	S	D	R	K	A	A	A	N
▲ 2.4	N	D	R	R	V	K	A	D
▼ 3.21	N	H	K	R	A	K	D	E
